# MiR-455-5p upregulation in umbilical cord mesenchymal stem cells attenuates endometrial injury and promotes repair of damaged endometrium via Janus kinase/signal transducer and activator of transcription 3 signaling

**DOI:** 10.1080/21655979.2021.2006976

**Published:** 2021-12-14

**Authors:** Dongyan Sun, Zhihe Jiang, Yanling Chen, Di Shang, Pan Miao, Jian Gao

**Affiliations:** a Department of Gynecology, Maternity and Child Health Care Hospital of Hubei Province, Wuhan, Hubei, China; b Department of Obstetrics and Gynecology, School of Medicine, Wuhan University of Science and Technology, Wuhan, Hubei, China; c Yangtze University Health Science Center, Jingzhou, Hubei, China

**Keywords:** Intrauterine adhesions, miR-455-5p, umbilical cord mesenchymal stem cells, SOCS3, JAK/STAT3

## Abstract

Umbilical cord mesenchymal stem cells (UCMSCs) are regarded as an ideal source for clinical use. Increasing evidence has suggested that microRNAs (miRNAs) work as a crucial regulator in the development of plentiful diseases, including intrauterine adhesions (IUA). Herein, we investigated the specific impacts of UCMSCs overexpressing miR-455-5p in IUA. UCMSCs were cocultured with endometrial stromal cells (ESCs). Thirty-two female mice were divided into four different treated groups: sham, model, model + UCMSC-miR-NC and model + UCMSC-miR-455-5p. Mice in model groups were induced by uterine curettage. MiR-455-5p overexpressed UCMSCs facilitated the proliferation and cell cycle progression of ESCs according to 5-ethynyl-2′-deoxyuridine assay and flow cytometry analysis. Hematoxylin-eosin and Masson staining revealed that miR-455-5p upregulation in UCMSCs increased the number of endometrial glands and suppressed endometrial fibrosis in murine uterine tissues. Western blotting displayed that miR-455-5p overexpressed UCMSCs promoted the activation of Janus kinase/signal transducer and activator of transcription 3 (JAK/STAT3) signaling in ESCs and murine uterine tissues. Mechanistically, miR-455-5p targeted 3’ untranslated region of suppressor of cytokine signaling 3 (SOCS3), which was confirmed by luciferase reporter assay. Reverse transcription quantitative polymerase chain reaction demonstrated that miR-455-5p was lowly expressed and SOCS3 was highly expressed in murine uterine tissues of IUA model. Moreover, Pearson correlation analysis showed that their expression was inversely correlated. Rescue assays suggested that inhibiting JAK/STAT3 signaling reversed effects of miR-455-5p on the behaviors of ESCs. The results indicated that miR-455-5p overexpression in UCMSCs helps to attenuate endometrial injury and repair damaged endometrium by activating SOCS3-mediated JAK/STAT3 signaling.

## Introduction

Intrauterine adhesions (IUA), also known as Asherman’s syndrome, results from damage to the basal layer of the endometrium [1]. Patients with moderate-to-severe IUA may struggle with pelvic pain, abnormal menstruation, recurrent miscarriages and even infertility [2,3]. IUA was reported to be the most common cause of uterine infertility, with approximately 25%–30% of infertile women suffering from IUA [4]. Hysteroscopy has been regarded as the mainstay for diagnosis of IUA and also as the most commonly used approach for IUA treatment followed by postoperative management [5]. Despite alternative strategies have been applied to limit or prevent re-adhesion, the recurrence rate after surgery is still very high, with 23% for moderate adhesion and 62% for severe adhesion [6]. Hence, more effective methods for IUA treatment are required to be found out.

Mesenchymal stem cells (MSCs) are multipotent stromal cells from adult tissues that have the capability of differentiating in different cell lineages [7]. Umbilical Cord MSCs (UCMSCs) is one of the ideal sources of MSCs, which are attractive for clinical use [8,9]. Compared to other kinds of MSCs, UCMSCs possess a superior capacity in differentiation, migration and self-renewal and can be invasively collected [10]. Increasing evidence has indicated that UCMSCs contribute to the repair of damaged endometrium [11]. For example, UCMSC transplantation restores endometrial thickness and attenuates excessive fibrosis in a rat model [12]. UCMSCs enhance the response of endometrium to hormones and improve endometrial proliferation as well as angiogenesis [13]. Furthermore, endometrial stromal cells (ESCs) function as a crucial cellular component in the endometrium, which is indispensable for proper physiological activities of the endometrium [14]. ESCs are implicated in the implantation and maintenance of pregnancy [15].

MicroRNAs (miRNAs) are small noncoding RNAs (21–24 nucleotides) which work as a critical post-transcriptional gene regulator [16]. Increasing evidence has revealed that miRNAs have great potential to be promising biomarkers with clinical utility [17]. Dysregulation of miRNAs are strongly associated with various human diseases, including IUA [18,19]. Recently, miR-455-5p has attracted increasing attention and has been reported to participate in multiple disorders. For example, miR-455-5p upregulation is related to the poor prognosis of patients with neonatal sepsis [20]. Moreover, miR-455-5p inhibits the proliferation and migration of vascular smooth muscle cells [21]. These suggest that miR-455-5p might be a biomarker in many diseases. Importantly, an IUA cell model was established in a previous study by treating ESCs with TGF-β1 [22]. ESCs were isolated from tissues of rats, and it was showed that rno-miR-455-5p overexpression could promote the proliferation of TGF-β1-treated ESCs [22]. However, the potential mechanism of hsa-miR-455-5p in IUA remains unanswered and there is no available evidence for the role of miR-455-5p in regulating the pathogenesis of IUA *in vivo*. Additionally, it has been indicated that miR-455-5p exerts its regulatory functions in some biological processes by targeting suppressor of cytokine signaling 3 (SOCS3) [23,24]. SOCS3 encodes a gene that can bind to Janus kinase 2 (JAK2) and inhibit the activity of JAK2 kinase [25]. Furthermore, SOCS3 is the main inhibitor of the Janus kinase/signal transducer and activator of transcription 3 (JAK/STAT3) signaling pathway [26]. The JAK/STAT3 signaling pathway has been reported to be implicated in the fibrosis of diseases, such as cardiac fibrosis, subepithelial fibrosis, and hepatic fibrosis [27–29].

This study intended to figure out the role and mechanism of miR-455-5p in UCMSC-treated ESCs and a IUA mouse model. We hypothesized that miR-455-5p might impact the proliferation and cell cycle progression of ESCs as well as the repair of damaged endometrium by targeting certain downstream gene and regulating the signaling pathway. This study might provide new ideas for the treatment of IUA.

## Materials and methods

### Identification of UCMSCs

Human Umbilical Cords were collected from healthy full-term deliveries in accordance with the Institutional Ethics Committee of Wuhan University of Science and Technology (Hubei, China). UCMSCs were isolated as previously described [30]. The isolated UCMSCs were cultured in Dulbecco’s Modified Eagle’s Medium (DMEM, Corning, Corning, NY, USA) containing 10% fetal bovine serum (FBS, Corning) and 1% penicillin-streptomycin (Corning) with 5% CO_2_ at 37°C until the confluency reached 90%. The cells at the third passage were used for further experiments. For differentiation potential assessment, UCMSCs (6 × 10^4^) were inoculated into a 24-well plate and cultured in adipogenic medium for 14 days and osteogenic medium for 21 days. Afterward, the cells were fixed with 4% PFA. After incubation for 30 min at room temperature, the cells were stained with Oil Red O or Alizarin Red solution (Santa Cruz, Dallas, TX, USA). Cell observation was achieved using a phase-contrast microscope (Nikon, Tokyo, Japan). For detection of MSC‐specific markers, UCMSCs were trypsinized with 0.25% trypsin-EDTA. Then cell suspensions were stained with phycoerythrin (PE)- or fluorescein isothiocyanate (FITC)-conjugated antibodies against human CD73 (ab157335), CD90 (ab95700), CD105 (ab18278), CD19 (ab52056), CD34 (ab223930), CD45 (ab214501), CD11b (ab213186) and HLA‐DR (ab77237) (dilution, 1:500; all from Abcam, Cambridge, MA, USA) for 30 min at room temperature. Then the cells were fixed with 1% paraformaldehyde in phosphate buffer saline (PBS). Subsequently, flow cytometry analysis was applied using the caliber cytometer FACS (Becton Dickinson, San Diego, CA, USA) [31].

### Cell transfection

MiR-455-5p mimics or negative control (miR-NC) (40 nM) obtained from GenePharma (Shanghai, China) were transfected into UCMSCs to upregulate miR-455-5p. Lipofectamine 2000 (Invitrogen, Carlsbad, CA, USA) was utilized for cell transfection following the manufacturer’s protocols [19]. Measurement of the transfection efficiency was conducted by RT-qPCR after 48 h.

### Coculture of UCMSCs and ESCs

ESCs were obtained from Procell Life Science&Technology (Wuhan, China; CP-H208) and were observed under a phase-contrast microscope (Nikon). ESCs were cocultured with DMEM, UCMSC-miR-NC or UCMSC-miR-455-5p mimics utilizing a Transwell system with a 0.4 μM pore polyester membrane (Corning) [32]. In brief, UCMSCs that had been previously transfected with miR-455-5p mimics or miR-NC were inoculated into 24-well plates (1 × 10^5^ cells/well). Simultaneously, corresponding ESC Transwell chambers (1.8 × 10^4^ cells/chamber) were seeded into the 24-well plates and cocultured with UCMSCs for 24 h. Then, the supernatant in the upper chambers was removed. ESCs were collected for subsequent experiments. Additionally, for inhibition of the JAK/STAT3 signaling, dimethyl sulfoxide (DMSO) containing JAK inhibitor AG490 (20 µM, Sigma, St. Louis, MO, USA) was used to culture ESCs.

### Reverse transcription quantitative polymerase chain reaction (RT-qPCR)

Total RNA was extracted from ESCs and murine uterine tissues utilizing TRIzol reagent (Invitrogen). The synthesis of cDNA for miRNA and mRNAs was achieved by reverse transcription of 1 µg RNA using miRNA First-Strand cDNA Synthesis kit (Sangon, Shanghai, China) and PrimeScript^TM^ RT reagent Kit (Takara, Dalian, China), respectively. RT-qPCR was then implemented with Power SYBR Green RT-PCR reagents (Applied Biosystems, Foster City, CA, USA). The relative gene expression of miR-455-5p and messenger RNAs (mRNAs) was normalized to U6 and GAPDH, respectively, and analyzed with the 2^−ΔΔCt^ method [33]. Primer sequences are listed in Table 1.

**Table 1.** Primer sequences used for RT-qPCR

Gene	Sequence (5’→3’)
Hsa-miR-455-5p forward	CGGTATGTGCCTTTGGACT
Hsa-mir-455-5p reverse	GTCGTATCCAGTGCAGGG
SOCS3 forward	AGAGCGGATTCTACTGGAGCG
SOCS3 reverse	CTGGATGCGTAGGTTCTTGGTC
MYLIP forward	GCAGGCGACTGGGAATCATAG
MYLIP reverse	CGGTTTCTCAGGTTTAGCCAT
TMEM30A forward	CGAAGGATGAAGTGGACGGT
TMEM30A reverse	AAACACGTTGCCCTCGATCT
TMED2 forward	GGTGACGCTTGCTGAACTGC
TMED2 reverse	AGATGAGGCCCATCTTGGTG
BRD1 forward	AACAAGGGTGGTGCCTTCAA
BRD1 reverse	ACCTCCTTCAGACTCACGGG
GAPDH forward	GAGTCAACGGATTTGGTCGT
GAPDH reverse	TTGATTTTGGAGGGATCTCG
U6 forward	CTCGCTTCGGCAGCACA
U6 reverse	AACGCTTCACGAATTTGCGT

### Western blotting

Total proteins were isolated from ESCs and uterine tissues using RIPA buffer (Beyotime, Shanghai, China) [34]. After quantification with a BCA assay kit (Thermo Fisher Scientific, Waltham, MA, USA) and separation by polyacrylamide gel electrophoresis, the proteins were transferred to polyvinylidene difluoride (PVDF) membranes (Invitrogen) which were subsequently blocked with 5% defatted milk. After that, the membranes were incubated at 4°C overnight with the following primary antibodies: anti-SOCS3 (ab280884, 1:1000), anti-β-actin (ab115777, 1:200), anti-p-JAK2 (ab32101, 1:1000), anti-JAK2 (ab108596, 1:5000), anti-p-STAT3 (ab267373, 1:1000), anti-STAT3 (ab68153, 1:1000) (all from Abcam) followed by being incubated with the horseradish peroxidase-conjugated secondary antibody of goat anti-rabbit IgG H&L (Abcam, ab6702, 1:1000) at room temperature for 2 h. The proteins were visualized using an ECL kit (Cwbiotech, Beijing, China) and quantified with the Odyssey infrared imaging system (LI-COR Biosciences, Lincoln, NE, USA).

### 5-ethynyl-2′-deoxyuridine (EdU) assay

KeyFluor488 Click-iTEdU kit (KeyGEN, Nanjing, China) was used for analysis of cell proliferation [35]. ESCs were seeded in 96-well plates (5 × 10^3^ cells/well). Forty-eight hours later, EdU labeling media (50 μM) was added to the plates and maintained for 2 h under 5% CO_2_ at 37°C. Then the cells were fixed with 4% polyformaldehyde containing PBS. DAPI was used to label cell nuclei. Images were observed using a fluorescence microscopy (Nikon).

### Flow cytometry analysis

To analyze cell cycle, ESCs in each treatment group were harvested and fixed in 70% ethanol. Then the cells (1 × 10^6^) were washed with PBS and incubated with PI/RNase Staining Buffer (BD Biosciences, San Jose, CA, USA) in the dark at 37°C. The distribution of cell cycle was analyzed with a FACSCanto II flow cytometer (BD Biosciences) [36].

### Luciferase reporter assay

The fragment of wild type or mutant SOCS3 3’untranslated region (3’UTR) containing predicted binding site of miR-455-5p was synthesized and subcloned into pmirGLO vectors (Promega, Madison, WI, USA) to establish SOCS3-Wt/Mut. Phusion Site-Directed Mutagenesis Kits (Thermo Fisher Scientific) were used to mutate the predicted binding site. The above vectors were then co-transfected with miR-455-5p mimics or miR-NC into ESCs using Lipofectamine 2000 (Invitrogen). The luciferase activity was measured with a dual luciferase® reporter assay system (Promega) after 48 h of co-transfection [37].

### Animal models

All the animal experiments were approved by the institutional Animal Care and Use Committee of Wuhan University of Science and Technology (Hubei, China) and were conducted strictly following the National Research Council Guide for the Care and Use of Laboratory Animals. Female six-week-old mice were purchased from Wuhan University of Science and Technology (Hubei, China). The establishment of IUA models was implemented according to the previous study [38,39]. Briefly, the mice were randomly divided into four groups: sham group, model group, model + UCMSC-miR-NC group and model + UCMSC-miR-455-5p mimics group (n = 8 per group). After anesthesia by intraperitoneal injection of 1% sodium pentobarbital (0.5 mL/kg), the abdominal cavity was opened to expose the uterus. Then, an incision was made at the bilateral uterus junctions. A mini-endometrial curette was inserted into uterus cavity through the incision for scraping until the uterus was hyperemic to the naked eye. After that, the abdominal cavity was closed. Mice in the sham group had the same laparotomy without any treatment for the uteri. In the model + UCMSC-miR-NC group and model + UCMSC-miR-455-5p mimics group, UCMSCs transfected with miR-NC or miR-455-5p mimics were injected into the mice, respectively. After treatment for 4 weeks, the mice were sacrificed by cervical dislocation, and uterine tissues were harvested.

### Hematoxylin-eosin (HE) staining and Masson staining

Murine uterine tissues were fixed with 4% paraformaldehyde for 24 h before paraffin embedding. After being deparaffinized and rehydrated, the slides were stained with hematoxylin and eosin. The pathological changes of uterine tissues were observed under a Leica DMLB2 microscope (Nussloch, Germany) [40]. The slides were subjected to Masson staining and four high-power fields were selected for each slide. The results of endometrial fibrosis were analyzed with Image-pro Plus 6.0 (Media Cybernetics, Inc., Bethesda, MD, USA).

### Statistical analysis

Data were analyzed using Statistical Product and Service Solutions (SPSS) 19.0 (IBM Corp, Armonk, NY, USA) and are expressed as the mean ± standard deviation (SD). Each experiment was repeated at least three times. Significant differences between two groups were examined by Student’s *t*-test, while those among more groups were evaluated by analysis of variance (ANOVA) followed by Tukey’s *post hoc* analysis [41]. The value of *p* < 0.05 was regarded as statistically significant.

## Results

MiR-455-5p was reported to exacerbate the process of IUA in a previous study; however, the specific impact and mechanism of miR-455-5p in UCMSCs underlying the progression of IUA are obscure. This study aimed to explore the role of miR-455-5p in UCMSCs-treated ESCs and murine IUA models. We hypothesized that miR-455-5p in UCMSCs might impact the phenotypes of ESCs and damaged endometrium by regulating the downstream target and signaling pathway. The results indicated that miR-455-5p upregulation in UCMSCs promotes the proliferation and cell cycle progression of ESCs, increases the number of endometrial glands and suppresses endometrial fibrosis in murine uterine tissues by activating SOCS3-mediated JAK/STAT3 signaling.

### Identification of UCMSCs

**Figure 1.**
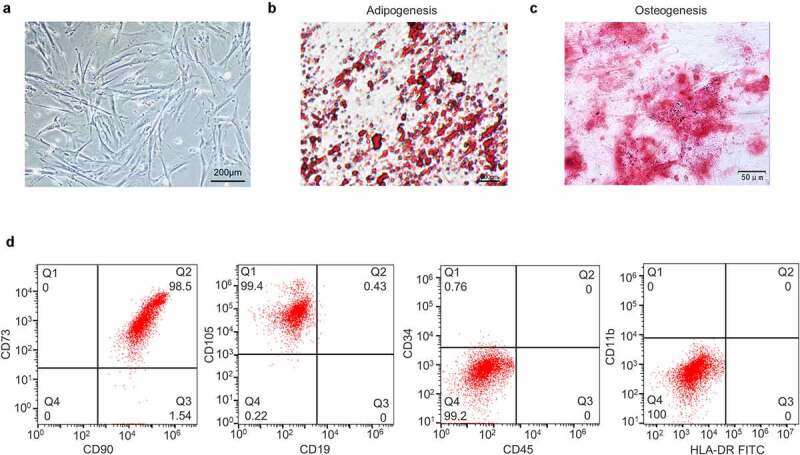
**Identification of UCMSCs**. (a) Morphology of UCMSCs. (b) Adipogenic induction of UCMSCs, detected by lipid droplets formation and Oil Red O staining. (c) Osteogenic induction of UCMSCs, detected by calcium nodule formation and Alizarin Red staining. (d) Detection of UCMSC surface markers by flow cytometry analysis

First, we identified UCMSCs from three aspects. Using phase-contrast microscopy, the cells were observed to show a fibroblast-like morphology, adherent growth and a spiral arrangement (Figure 1(a)). Afterward, differentiation induction media were used to examine the multipotency of UCMSCs. The vacuoles of cells cultured in adipogenic medium gathered together to form lipid droplets in cytoplasm and displayed Oil Red O staining (Figure 1(b)). In parallel, the cells cultured in osteogenic medium exhibited a calcium nodule shape and Alizarin Red staining (Figure 1(c)). Moreover, flow cytometry analysis indicated that the cells were positive for CD90, CD73 and CD105 (˃95%), while were negative for CD19, CD45, CD34, CD11b and HLA-DR (˂5%) (Figure 1(d)). The characteristics of cells from three aspects were all in accord with MSCs.

### MiR-455-5p promotes cell proliferation and cell cycle progression

**Figure 2.**
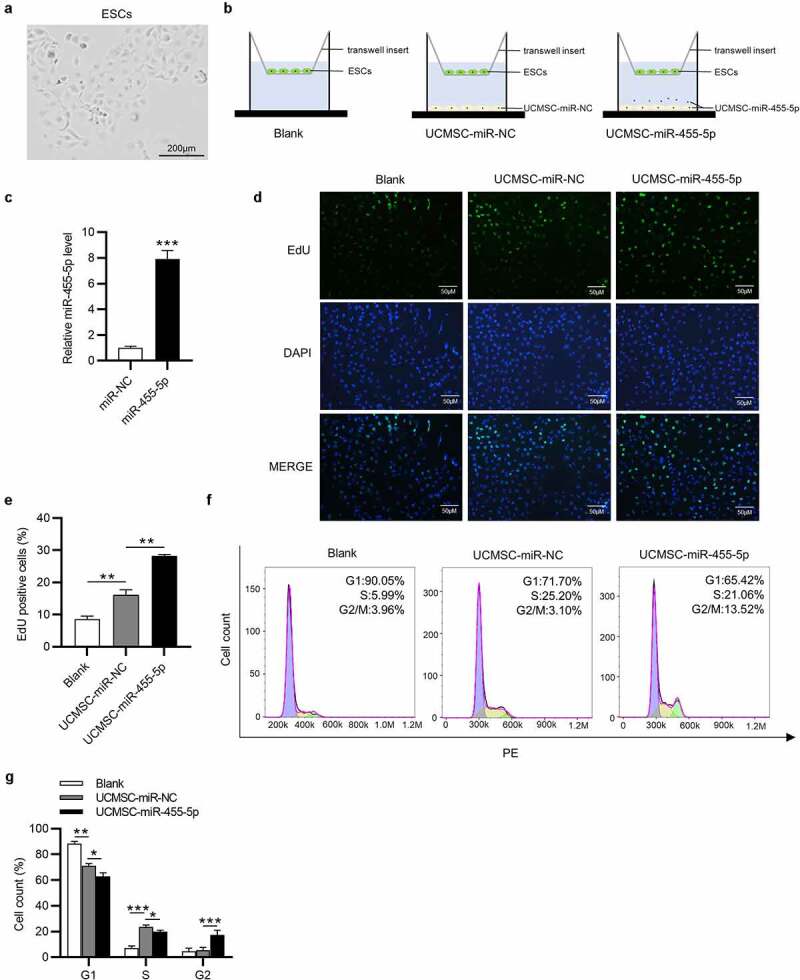
**MiR-455-5p contributes to cell proliferation and cell cycle progression**. (a) Morphology of ESCs by phase-contrast microscopy. (b) ESCs were cocultured with blank control medium, UCMSCs with miR-NC or UCMSCs with miR-455-5p. (c) RT-qPCR of miR-455-5p mimics transfection efficiency in UCMSCs. (d, e). EdU assay of the proliferation of ESCs in UCMSC-miR-NC, UCMSC-miR-455-5p mimics or the control groups. (f, g). Flow cytometry analysis of cell phase distribution of ESCs in above groups. *p < 0.05, **p < 0.01, ***p < 0.001

The primary ESCs were observed to adhere to the plastic culture dishes and show a spindle-shaped and fibroblast-like morphology (Figure 2(a)). To investigate the impact of miR-455-5p in UCMSC-treated ESCs, we cocultured ESCs with blank control medium, UCMSCs with miR-NC or UCMSCs with miR-455-5p mimics in a Transwell system (Figure 2(b)). After coculture, ESCs were collected from Transwell inserts for further experiments. Then, we detected transfection efficiency of miR-455-5p mimics in UCMSCs and as shown by RT-qPCR, miR-455-5p level was obviously raised after transfection of miR-455-5p mimics (Figure 2(c)). Afterward, EdU assay was implemented to examine impact of miR-455-5p on cell proliferation. Compared to the control group, UCMSC-miR-NC group showed an increased number of EdU positive cells, while UCMSC-miR-455-5p mimics group displayed a higher level of EdU positive cells (Figure 2(d,e)). Furthermore, a similar trend was observed in flow cytometry analysis. The number of ESCs at S phase evidently increased in UCMSC-miR-NC group, proving the promotive influence of UCMSCs on cell cycle (Figure 2(f,g)). Additionally, miR-455-5p mimics evidently induced an increase in the G2 phase cell cycle populations (Figure 2(f,g)), suggesting that miR-455-5p promoted S/G2 transition in ESCs. Hence, the above results indicate that miR-455-5p facilitates the proliferation and cell cycle progression of ESCs cocultured with UCMSCs.

### MiR-455-5p attenuates endometrial injury and promotes repair of damaged endometrium

**Figure 3.**
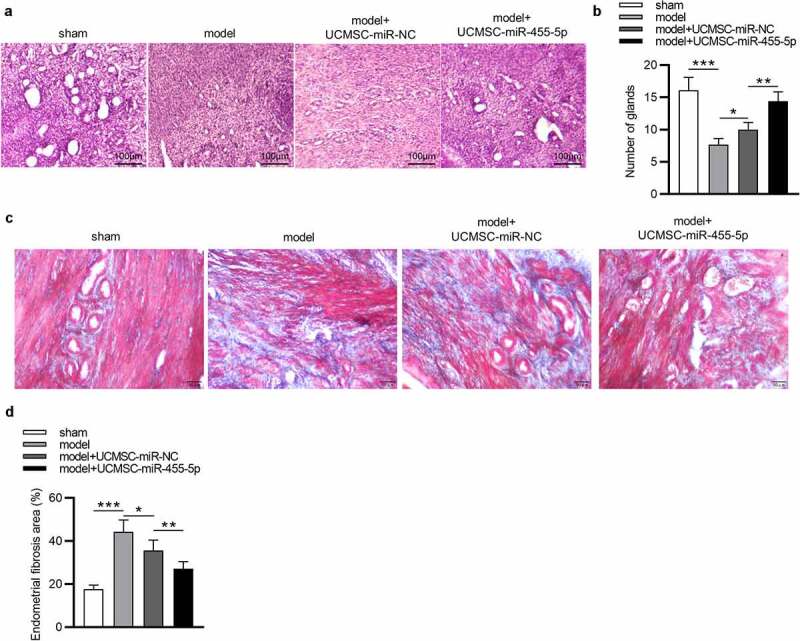
**MiR-455-5p alleviates endometrial injury and promotes repair of damaged endometrium**. (a, b) HE staining for detecting the impact of miR-455-5p on the number of endometrial glands. (c, d) Masson staining for evaluating the influence of miR-455-5p on endometrial fibrosis. *p < 0.05, **p < 0.01, ***p < 0.001

To verify whether miR-445-5p affects the progression of IUA, we established a murine model of IUA and collected the uterine tissues at the 4^th^ week after modeling. As revealed by HE staining, in the sham group, the uterine cavity surface was covered with columnar epithelium and the endometrial glands were abundant, arranged in round or oval shapes (Figure 3(a,b)). In the model group, tissues were observed to be adherent, and the number of glands was markedly decreased (Figure 3(a,b)). In comparison to the model group, the number of glands in the model + UCMSC-miR-NC group displayed was elevated (Figure 3(b)). Notably, miR-455-5p treatment significantly led to an enhancement in the number of glands compared with the model + UCMSC-miR-NC group (Figure 3(b)). These results indicated that both UCMSCs and miR-455-5p could increase the number of glands. Furthermore, Masson staining was performed to investigate the impact of miR-455-5p on endometrial fibrosis. As shown by the results, compared to that in the sham group, the fibrotic area in the endometrium of model group was remarkably enhanced, but UCMSC treatment resulted in a slight reduction in fibrosis (Figure 3(c,d)). Notably, an evident reduction in fibrotic area was displayed in miR-455-5p mimics-treated group compared with the miR-NC-treated group (Figure 3(c,d)), suggesting that miR-445-5p contributed to the improvement of endometrial fibrosis. Collectively, miR-455-5p increases the number of endometrial glands and suppresses endometrial fibrosis, thereby helping to attenuating endometrial injury and promoting repair of damaged endometrium.

### MiR-455-5p directly targets SOCS3

**Figure 4.**
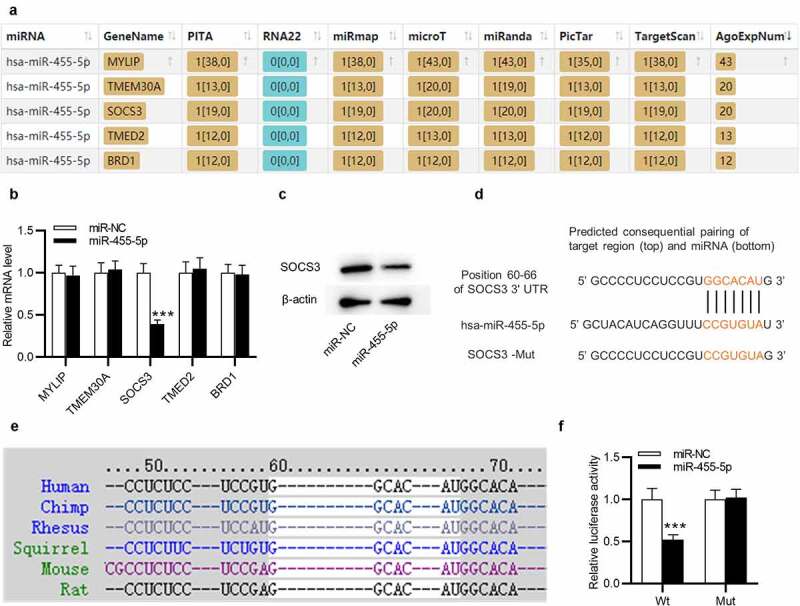
**MiR-455-5p targets SOCS3 in ESCs**. (a) The downstream targets of miR-455-5p predicted by ENCORI with the condition of PITA, miRmap, microT, miRanda, PicTar, TargetScan and AgoExpNum>10. (b) RT-qPCR analysis of the mRNA levels in ESCs after transfection of miR-455-5p mimics. (c) Western blotting of SOCS3 protein expression in ESCs with above transfection. (d) The complementary site of has-miR-455-5p on SOCS3 3’UTR predicted by TargetScan. (e) The above binding site is highly conserved in multiple species. (f) A luciferase reporter assay for examining the interaction between SOCS3 and miR-455-5p. ***p < 0.001

Next, we examined the regulatory mechanism of miR-445-5p in ESCs. Five downstream targets of miR-455-5p were predicted by ENCORI (http://starbase.sysu.edu.cn/) with the screening condition of predicted program: PITA, miRmap, microT, miRanda, PicTar, TargetScan and number of supported AGO CLIP-seq experiments (AgoExpNum)>10 (Figure 4(a)). Afterward, RT-qPCR was utilized to evaluate the levels of the five candidate mRNAs in ESCs transfected withmiR-455-5p mimics. Only SOCS3 level was markedly reduced by overexpressed miR-445-5p in ESCs (Figure 4(b)). Additionally, SOCS3 protein level was observed to be decreased by miR-455-5p mimics (Figure 4(c)). The complementary site of hsa-miR-445-5p on SOCS3 is confirmed by TargetScan (http://www.targetscan.org/) (Figure 4(d)). Importantly, TargetScan suggests that this binding site is highly conserved in multiple species, including mouse (Figure 4(e)). The interaction between miR-455-5p and SOCS3 was further confirmed by a luciferase reporter assay. The luciferase activity of SOCS3-Wt was obviously weakened by miR-455-5p mimics in ESCs, while that of SOCS3-Mut almost stayed the same (Figure 4(f)). Thus, SOCS3 is targeted by miR-455-5p in ESCs.

### MiR-455-5p expression is negatively correlated with SOCS3 expression in murine uterine tissues

**Figure 5.**
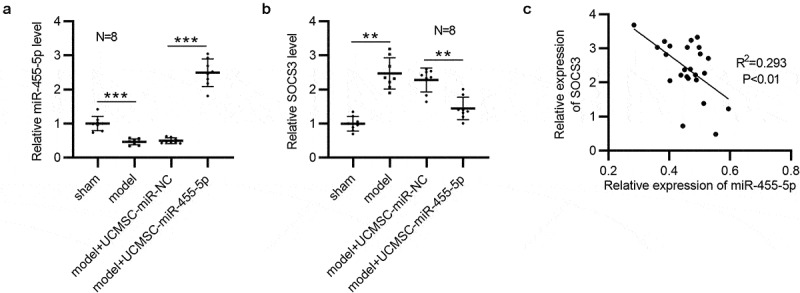
**MiR-455-5p is inversely correlated with SOCS3 in murine uterine tissues**. (a) RT-qPCR analysis of miR-455-5p levels in the four groups (n = 8/group). (b) RT-qPCR analysis of SOCS3 levels in the four groups (n = 8/group). (c) Pearson correlation analysis for analyzing the expression correlation between SOCS3 and miR-455-5p. **p < 0.01, ***p < 0.001

The expression correlation between miR-455-5p and SOCS3 was further analyzed. Obviously, in comparison to that in the sham group, the level of miR-455-5p was reduced in the model group (n = 8/group), as shown by RT-qPCR (Figure 5(a)). A higher level of miR-455-5p was observed in UCMSC-miR-455-5p mimics-treated group than that in UCMSC-miR-NC-treated group (n = 8/group) (Figure 5(a)). Moreover, SOCS3 displayed a higher level in the model group than it was in the sham group, and SOCS3 was significantly downregulated in UCMSC-miR-455-5p mimics-treated group compared to UCMSC-miR-NC-treated group (n = 8/group) (Figure 5(b)). Additionally, Pearson correlation analysis suggested that miR-455-5p expression had a negative correlation with SOCS3 expression in murine uterine tissues (n = 24) (Figure 5(c)).

### MiR-455-5p activates the JAK/STAT3 signaling pathway

**Figure 6.**
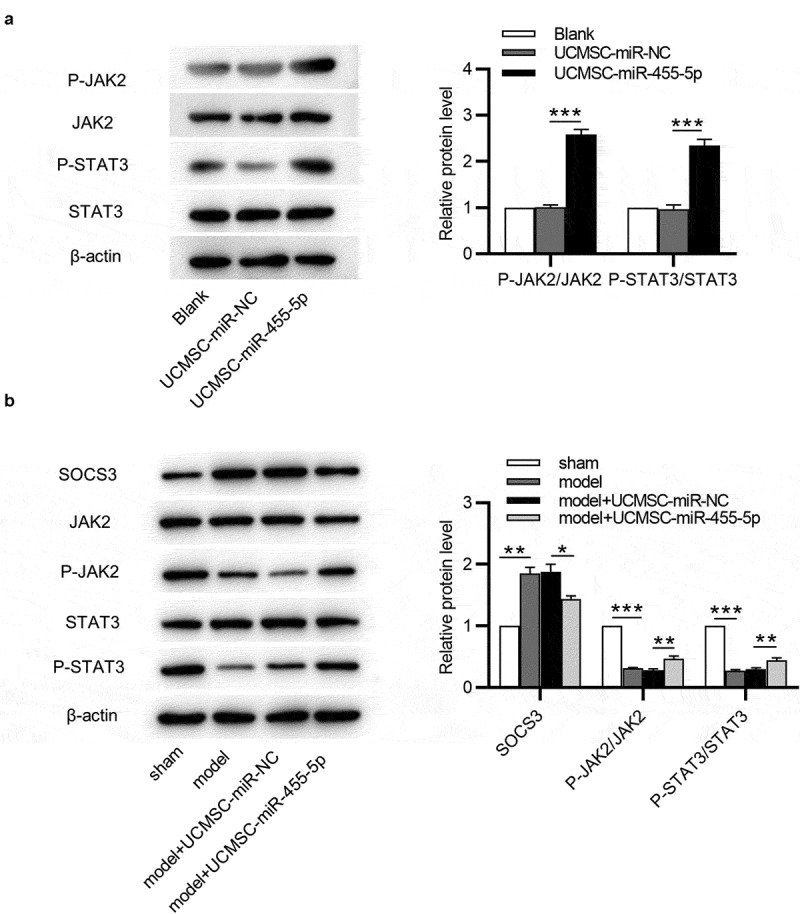
**MiR-455-5p activates the JAK/STAT3 signaling pathway**. (a) Western blotting for evaluating the levels of the JAK/STAT3 signaling pathway-associated proteins in ESCs. (b) Western blotting for assessing the levels of above proteins in murine uterine tissues. *p < 0.05, **p < 0.01, ***p < 0.001

SOCS3 is known to be an inhibitor of the JAK/STAT3 signaling pathway [26]. Subsequently, we investigated how miR-455-5p regulated the SOCS3-mediated JAK/STAT3 signaling pathway. Western blotting was used for evaluating the levels of JAK/STAT3 signaling pathway-associated proteins. As shown by the results, miR-455-5p mimics remarkably enhanced protein levels of phosphorylated (p)-JAK2 and p-STAT3 in ESCs (Figure 6(a)). Additionally, SOCS3 protein expression was obviously upregulated in the model group and model + UCMSC-miR-NC group and was slightly upregulated in miR-455-5p mimics-treated group (Figure 6(b)). With respect to those in the sham group, the protein levels of p-JAK2 and p-STAT3 were reduced in the model and model + UCMSC-miR-NC groups, and this effect was reversed after treatment of miR-455-5p mimics (Figure 6(b)). Hence, miR-455-5p facilitates the phosphorylation of JAK2 and STAT3 by targeting SOCS3 in ESCs and endometrial tissues, thereby activating the JAK/STAT3 signaling pathway.

### The influence of miR-455-5p on the phenotypes of ESCs is reversed by AG490

**Figure 7.**
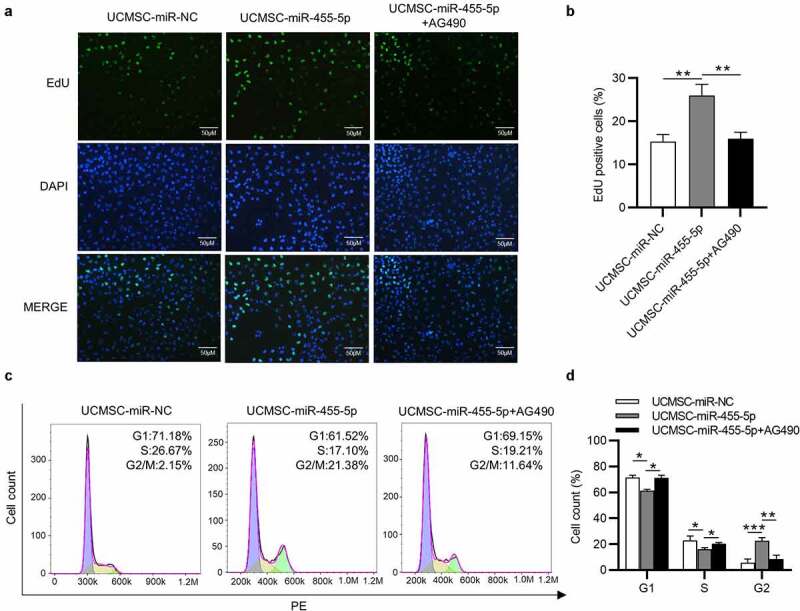
**AG490 treatment reverses the influence of miR-455-5p on the phenotypes of ESCs**. (a, b) EdU assay for examining the proliferation of ESCs treated with UCMSC-miR-NC, UCMSC-miR-455-5p mimics or UCMSC-miR-455-5p mimics +AG490. (c, d) Flow cytometry analysis of cell cycle progression of ESCs with above treatment. *p < 0.05, **p < 0.01, ***p < 0.001

To further elucidate that miR-455-5p influences the phenotypes of ESCs by regulating JAK/STAT3 signaling, AG490 was used to inhibit the JAK/STAT3 signaling pathway. EdU assays demonstrated that the proliferation of ESCs promoted by miR-455-5p was attenuated after AG490 treatment (Figure 7(a,b)). Likewise, flow-cytometry analysis indicated that miR-455-5p mimics promoted the cell cycle progression of ESCs, but this effect was reversed by AG490, which led to the decrease of cell cycle at G2/M (Figure 7(c,d)). Thus, the inactivation of JAK/STAT3 signaling induced by AG490 reverses the influence of miR-455-5p on the phenotypes of ESCs.

## Discussion

One of the most important pathological process of IUA is endometrial fibrosis [42]. Considerable studies have demonstrated that MSCs exert an indispensable role in IUA. For example, MSC treatment suppresses inflammation and fibrosis in a rat model, thereby improving the condition of IUA [43]. Moreover, MSCs were reported to be a therapeutic agent of endometrial fibrosis [44]. MSCs are able to limit endometrial fibrosis via inhibiting TGF-β that is involved in the formation of IUA [44]. UCMSCs, one of the best sources of MSCs, was once reported to ameliorate the severity of liver fibrosis [45]. Moreover, UCMSCs contribute to endometrial regeneration and repair [11]. In the present study, we cocultured ESCs with UCMSCs and confirmed that UCMSCs could promote the proliferation and cell cycle progression of ESCs and help to repair the damaged endometrium.

Emerging evidence has suggested that miRNAs are associated with a multitude of human disorders [46]. MiR-455-5p has been reported to be a potential biomarker in many diseases. For example, miR-455-5p upregulation influences retinol absorption in a lung hypoplasia model by regulating STRA6 [47]. Moreover, miR-455-5p is considered to be a diagnostic and prognostic marker for neonatal sepsis [20]. A previous study has indicated that rno-miR-455-5p contributes to the proliferation of ESCs that are treated with TGF-β for establishing an IUA cell model [22]. MiR-455-5p was reported to be involved in the regulation of cell cycle in multiple diseases. For example, miR-455-5p is sponged by HOXA-AS3 in atherosclerosis and can reverse HOXA-AS3 knockdown-mediated promotive effect on cell cycle [48]. In the current study, miR-455-5p was overexpressed in UCMSCs that were cocultured with ESCs or implanted into the mice. It was found that miR-455-5p overexpression facilitated the proliferation and cell cycle progression of ESCs. Additionally, it was shown that after overexpressing miR-455-5p, the number of endometrial glands was increased and endometrial fibrosis was limited in murine uterine tissues, suggesting that miR-455-5p was favorable for the regeneration and repair of damaged endometrium.

MiRNAs are recognized to regulate the stability and translation of mRNAs by targeting their 3’UTR [49]. Bioinformatics tool ENCORI was utilized for predicting the downstream targets containing binding site for miR-455-5p, and SOCS3 was singled out. SOCS3, a member of SOCS family, was reported to affects the fibrosis of some diseases. For example, absence of SOCS3 leads to the liver fibrosis by increasing the production of TGF-β that was mediated by STAT3 [50]. In our study, a series of assays were implemented to verify the interaction between SOCS3 and miR-455-5p. Notably, miR-455-5p expression was shown to have a negative correlation with SOCS3 expression in murine uterine tissues. Furthermore, previous investigations have elucidated that SOCS3 works as an inhibitor of the JAK/STAT3 signaling pathway [26,51]. Here, we investigated the impact of miR-455-5p on SOCS3-mediated JAK/STAT3 signaling pathway. It was found that miR-455-5p downregulated SOCS3 and facilitated the phosphorylation of JAK2 and STAT3 in ESCs and murine uterine tissues, indicating that miR-455-5p exerted its influences by activating the JAK/STAT3 signaling. To further confirm this effect, rescue assays were carried out. AG490 was used as an inhibitor of the JAK/STAT3 signaling pathway. As anticipated, after inhibiting the JAK/STAT signaling, the promotive influence on the proliferation and cell cycle progression of ESCs induced by miR-455-5p was attenuated.

## Conclusion

In conclusion, this study explored the functions and mechanism of miR-455-5p overexpressed UCMSCs in the proliferation and cell cycle progression of ESCs. Moreover, we investigated the impacts of miR-455-5p on endometrial glands and endometrial fibrosis in a mouse model. Overexpression of miR-455-5p in UCMSCs promotes ESC proliferation and cell cycle progression, attenuates endometrial injury and facilitates repair of damaged endometrium by regulating SOCS3-mediated JAK/STAT3 signaling pathway. These findings might provide new ideas for treating IUA.
